# High-Fat-Diet-Induced Hyperglycemia Alters Liver Extracellular Matrix Composition in Mice Model

**DOI:** 10.3390/cells15121105

**Published:** 2026-06-18

**Authors:** Roza Izgilov, Nahum Kavin, Omri Ofek, Nadav Kislev, Dafna Benayahu

**Affiliations:** Department of Cell and Developmental Biology, Gray Faculty of Medical and Health Sciences, Tel Aviv University, Tel Aviv 6997801, Israel

**Keywords:** liver tissue, extracellular matrix, high-fat diet, AGEs, insulin resistance, ECM remodeling

## Abstract

**Highlights:**

**What are the main findings?**

- High-fat-diet (HFD) nutrition alters the biochemical and physical properties of liver ECM.
- HFD-induced glycation leads to protein modifications.

**What is the implication of the main finding?**

- SEM-CL and advanced imaging reveal nanoscale changes in liver ECM.

**Abstract:**

Regenerative medicine integrates interdisciplinary approaches towards restoring the function of diseased organs. This study examined alterations that occurred in the liver under a high-fat diet (HFD) with the development of obesity and fatty liver, and changes in metabolic homeostasis and glucose levels, in mice. HFD nutrition causes hyperglycemia, leading to the formation and accumulation of advanced glycation end-products (AGEs) promoting protein post-translational modifications (PTMs) and introducing crosslinking in the extracellular matrix (ECM). Using histological and gene expression analyses, we detected an increase in adiposity, as well as in ECM protein deposition in the liver. Further, decellularization of the liver yielded the isolated ECM organ scaffold, allowing us to analyze the chemical modification in proteins by various imaging methods combined with spectroscopy. The measurements of intrinsic protein fluorescence are consistent with increased AGE-associated levels. SEM allows for the visualization of ECM fiber thickening as a result of protein crosslinking. Using cathodoluminescence, a label-free imaging method, we confirmed the protein modifications. The combination of innovative technologies highlights the ECM structural alterations associated with impaired glucose regulation and liver adiposity. These findings provide novel views on liver-scaffold ECM structure under metabolic diseases that will play a significant role in accelerating the understanding of effective regenerative therapies.

## 1. Introduction

The extracellular matrix (ECM) is a complex assembly of proteins that forms the structural scaffold of organs. ECM macromolecules have a slow remodeling rate, being affected by nutrition and metabolism through post-translational modifications (PTMs) [1]. Nutrition, particularly a high-fat diet (HFD), promotes hyperglycation. The excessive glucose generates highly reactive metabolites that induce glycation adducts, leading to PTMs, ultimately altering protein structure and function [2,3,4]. Modified ECM macromolecules alter their structure and consequently affect cell signaling, leading to changes in tissue functionality [3,5,6,7] and regeneration potential, and contributing to associated pathologies [8,9,10]. ECM proteins form a complex micro-environmental niche for cells; therefore, the ECM alterations modify structural support to cells and thereby affect their function [11,12].

A high-fat diet is a widely used model for inducing metabolic dysfunction in rodents, recapitulating key features of human obesity, insulin resistance, and hepatic steatosis [4,5,13]. Consumption of HFD promotes ectopic lipid accumulation in the liver, impairing mitochondrial function and dysregulating lipogenic signaling [3,14], leading to hepatic steatosis. This condition is associated with elevated circulating glucose, creating a pro-glycation environment in which sugars react non-enzymatically with ECM proteins to form AGEs [1,15].

The profound impact of HFD nutrition led us to investigate the effects of induced hyperglycation on liver ECM remodeling and regeneration in this study. In a previous study, we reported that HFD nutrition induces changes in adipose tissue ECM proteins, which was demonstrated by various histological staining [16]. Here, we focused on the isolation and analysis of liver ECM protein modifications via glycoxidation, which produced carbonyl intermediates and radicals, forming direct covalent modifications on ECM proteins. Carbonylation is a hallmark of oxidative stress, and Amadori products result in the formation of advanced glycation end-products (AGEs). Thus, oxidative damage to proteins is manifested by carbonyl-group additions, resulting in the generation of AGEs [14,15,16].

We histologically analyzed the liver cells and ECM in a HFD metabolic mouse model, as well as the decellularized ECM (dECM). The combination of confocal and scanning electron microscopy (SEM) with optical cathodoluminescence (CL) provided simultaneous acquisition in a scanning image and spatial mapping. Other confocal microscopies detected the fibrosis alterations through autofluorescence and immunostaining on the 2D and 3D levels of the ECM proteins. The technologies used allow us to present new views to confirm the protein alterations that underlie the nutritional effects on organ remodeling leading to pathophysiology. The importance of exploring ECM glycation lies in its potential to alter cell functionality and signaling pathways and therefore hamper organ regeneration. Glycation alterations of ECM are crucial for organ functionality, and a better understanding will lead us towards potential targets for clinical therapy. It is recognized that hepatic steatosis has been directly associated with dietary composition, with HFD driving AGE accumulation and ECM remodeling in both clinical and experimental settings [17].

## 2. Materials and Methods

**Mice:** Male 6-week-old C57BL/6J mice were fed with either a standard carbohydrate (CHO) or high-fat diet (HFD, 60% calories from fat, Research Diets, Inc., New Brunswick, NJ, USA) for 15 weeks and provided with water ad libitum. The mice were kept in a conventional facility with a 12 h light/dark cycle. Animal care and experiments followed the guidelines of the TAU-IACUC, approval number 01-21-044. Weight was measured after overnight fasting at six points in time during the 15 weeks. At sacrifice, livers were collected and immediately frozen at −80 °C for RNA isolation or fixed for histology staining and tissue decellularization for retrieving ECM (*N* = 5–9 and mentioned for each experiment).

**Glucose level and GTT:** The intra-peritoneal glucose tolerance test (IP-GTT) was performed and fasting glucose was measured at the end of the 15-week feeding period. The mice blood glucose level was measured after overnight fasting using a CONTOUR^®^ Plus glucometer (Ascensia Diabetes Care, Parsippany, NJ, USA) and test strips (Padagis™, Padagis LLC, Minneapolis, MN, USA). For GTT, after an overnight fasting, an IP injection of 20% *w/v* in D-Glucose (Sigma G-6138, Merck & Co., Inc., Rahway, NJ, USA) in PBS (1 g glucose to 1 kg weight) was performed for both control and HFD mice. Blood glucose was measured at the time of injection and at multiple times up to 120 min thereafter.

**Molecular expression, RNA isolation, and qPCR:** Total RNA was extracted from 50 mg liver using the Isolation Hybrid-R Kit (305-101, GeneAll^®^ Hybrid-R™, GeneAll Biotechnology Co., Ltd, Seoul, Korea), and reverse-transcribed with the UltrascriptTM cDNA Synthesis Kit (PB30.11-10, PCR Biosystems, Wayne, PA, USA). Transcript levels were quantified using qPCR amplified with SYBR green (Applied Biosystem, Waltham, MA, USA) using the StepOne plus system (Thermo Fisher Scientific, Waltham, MA, USA). Gene expression, measured using primers (Table 1), was normalized to the ribosomal protein lateral stalk subunit P0 (RPLP0) using the delta–delta Ct method [18].

**Histology staining and analysis:** Liver samples were fixed in 4% paraformaldehyde overnight at 4 °C; washed with PBS, series of ethanol, and xylene; and embedded into paraffin blocks. Sections of 5 µm thickness were de-paraffinized, rehydrated, and stained with hematoxylin–eosin (H&E) or picrosirius red [16]. Picrosirius red staining enabled the visualization of a bright red birefringent signal from collagen fibers, which was observed under a polarizing filter using light microscopy (Nikon Optiphot-2, Nikon, Tokyo, Japan). Stained sections were photographed under the Aperio slide scanner microscope (Aperio Versa 200; Leica, Teaneck, NJ, USA) and analyzed using Fiji ImageJ-Win64 software (NIH, Bethesda, MD, USA). Hepatic lipid content was quantified from H&E-stained sections using ImageJ software. Lipid vacuole area was expressed as a percentage of total field area. Steatosis, hepatocyte ballooning, and lobular inflammation were scored according to the NAFLD activity score (NAS) [19].

**Decellularization of liver tissue (dECM):** Liver tissue was decellularized through extensive washes under agitation in 0.1% ethylene–diamine–tetra-acetic acid (EDTA) for blood removal at room temperature (RT). Further, the samples underwent a series of washes in double-distilled water (DDW) for 24 h, followed by incubation in 0.1% ammonium hydroxide containing 1% Triton X-100 for 84 h with daily solution changes, and then washed again in DDW for 48 h [20,21].

**Immunofluorescence staining of ECM proteins:** Whole-mount liver sample staining was performed as earlier described [21]. In short, the samples were stained with primary antibodies: glucose transporter 4 (GLUT4; SC 53566), elastin (SC-166543), nidogen (SC-33706) (Santa Cruz Biotechnology, Dallas, TX, USA), and collagen type 1 (Coll 1; C2456, Sigma, Merck & Co., Inc., Rahway, NJ, USA). The secondary anti-mouse antibodies used were Alexa 488 (A21121), Alexa 555 (A-21127, Invitrogen, Thermo Fisher Scientific, Waltham, MA 02451, USA), Alexa 488 (1090-30), Alexa 555 (1080-32), and Alexa 647 (1071-31) (Southern Biotech, Birmingham, AL, USA). Lipid content was stained with 10 µg/mL Nile Red (Sigma N-3013). A Fluoroshield mounting medium containing 4′,6-diamidino-2-phenylindole (DAPI) (Electron Microscopy Sciences, 17985-10, Morgantown, PA, USA) was used for nuclei staining. The samples were viewed, and 2D images were acquired using an SP8 confocal microscope (Leica, Wetzlar, Germany). 3D image reconstruction was performed using a 3i Marianas spinning-disk confocal microscope (Intelligent Imaging Innovations, Denver, CO, USA). Imaris software was used for image analysis (Oxford Instruments, Concord, MA, USA).

**ECM autofluorescence properties:** Unstained dECM samples were scanned with white light lasers (WLLs) using a STELLARIS 5 Confocal Microscope. The WLL provides higher photon detection efficiency (PDE) with low dark noise using the HyD detector. Liver dECM sample autofluorescence measurements and ECM microscopy visualization, under an excitation of 485–573 nm for intrinsic autofluorescence emission, was followed by image analysis using LAS X Navigator software (Leica Microsystems, Wetzlar, Germany).

**Scanning electron microscope (SEM) cathodo-luminescence (CL) (SEM-CL):** The combined SEM-CL uses electron-induced photon emission to analyze native, unstained, and freeze-dried dECM within an extended wavelength range. The technique enables spatial 3D imaging and CL spectral analysis, with luminescence recorded. Liver dECM samples were frozen at −80 °C, then lyophilized using freeze-drying (CHRIST Alpha). Samples were imaged using a SPARC cathodoluminescence (Delmic, Delft, the Netherlands) Apreo 2S LoVac Scanning Electron Microscope (ThermoFisher, Waltham, MA, USA). The SEM was used in standard mode, reducing electric and magnetic fields between the sample and the pole piece following SPARC standard requirements. Obtained CL spectra were analyzed using ODEMIS software (https://www.delmic.com (accessed on 23 April 2026)).

**Statistical analysis and illustration:** Statistical analysis was conducted using GraphPad Prism 9 software (La Jolla, CA, USA). Sample size and statistical tests are presented in legends to figures, with a *p*-value < 0.05 as statistically significant: (ns) *p* ≥ 0.05; * *p* < 0.05; ** *p* < 0.01; *** *p* < 0.001; **** *p* < 0.0001. Data are presented as means ± SD.

**Schematic illustrations**: Created using the BioRender software site (https://biorender.com).

## 3. Results

**Figure 1:**
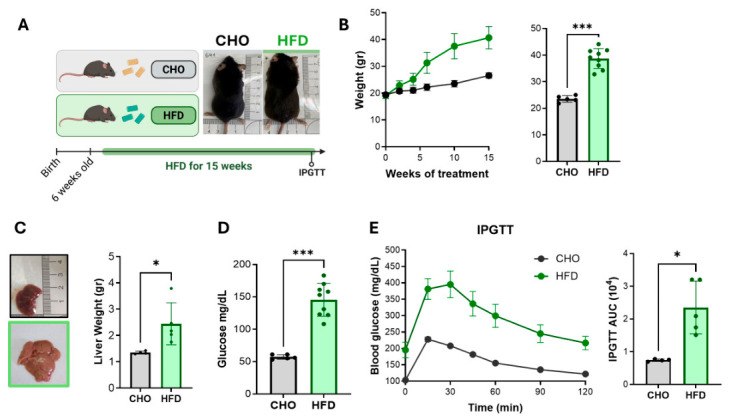
**Impact of HFD on weight and glucose metabolism in mice.** (**A**) Schematic illustration of the HFD mouse model with representative mouse images. Mice were fed either a control chow (CHO) diet or a HFD for 15 weeks. (**B**) Weight measurements during the 15-week feeding period (left) and endpoint body weight distribution (right) of mice fed a CHO diet or a HFD (n = 5–9 per group). Data presented as mean ± SD. Mann–Whitney U test, *** *p* < 0.001. (**C**) Liver weight measurement (n = 4–5 per group). Student’s *t*-test with Welch’s correction. * *p* < 0.05. (**D**) Fasting blood glucose measurement after overnight fasting (mean ± SD; n = 5–9 per group). Mann–Whitney test, *** *p* < 0.001. (**E**) Intraperitoneal glucose tolerance test (IP-GTT) performed after overnight fasting (left; mean ± SEM). GTT AUC quantification (right; mean ± SD; n = 4–5 per group). Student’s *t*-test with Welch’s correction, * *p* < 0.05.

Mice fed a high-fat diet (HFD) for 15 weeks (experimental design, Figure 1A) exhibited a progressive increase in body weight compared to those fed a standard carbohydrate (CHO) diet during the follow-up period (Figure 1B). Macroscopic examination of the livers from HFD-fed mice revealed an increase in liver mass and a yellowish appearance, indicative of lipid accumulation (Figure 1C). At the end of the study, we assessed the metabolic impact of HFD by measuring fasting glucose levels and saw a significant increase after overnight fasting (Figure 1D). A glucose tolerance test (GTT) further revealed marked differences between control and HFD mice (Figure 1E), with elevated glucose levels resulting from impaired glucose regulation, consistent with the onset of insulin resistance. This experiment complements our previous report, which provided a detailed analysis of metabolic alterations and changes in adipose tissue [16].

**Figure 2:**
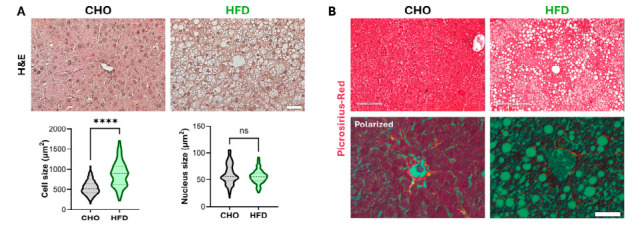
**Histological analysis of liver alterations following HFD in mice.** (**A**) Histological representation of liver tissue from control and HFD-treated mice, stained with H&E. Quantification of hepatocyte cells and nuclear size (scale bar = 200 µm). Student’s *t*-test with Welch’s correction (ns) *p* > 0.05; **** *p* ≤ 0.0001. (**B**) Picrosirius red staining highlights collagen fibers accompanied by a birefringence signal around the central vein (polarized light, lower panel, scale bar = 100 µm). Upper panel: scale bars = 100 μm (left) and 200 μm (right).

Histological analysis of the liver revealed that HFD-fed mice had increased adiposity and altered liver architecture compared to CHO-fed controls. In H&E-stained sections, hepatocytes from HFD mice presented a distinct accumulation of lipid droplets (Figure 2A). Quantification revealed an increase in hepatocyte cell size, while nuclear size remained unchanged (Figure 2A). From these histology sections, quantification of hepatic lipid content revealed a mean lipid accumulation of 32.89 ± 2.57% in HFD-fed mice, corresponding to a NAS steatosis grade of 2. Combined with a hepatocyte ballooning score at grade 1, the HFD samples received a total NAS of 3, indicating the progression of steatosis.

Picrosirius red staining allows for the visualization of collagen-rich areas (Figure 2B, upper panels). Under polarized light microscopy, visible collagen was observed around the central vein and between hepatocytes, indicative of fibrosis in response to HFD (Figure 2B, lower panels).

**Figure 3:**
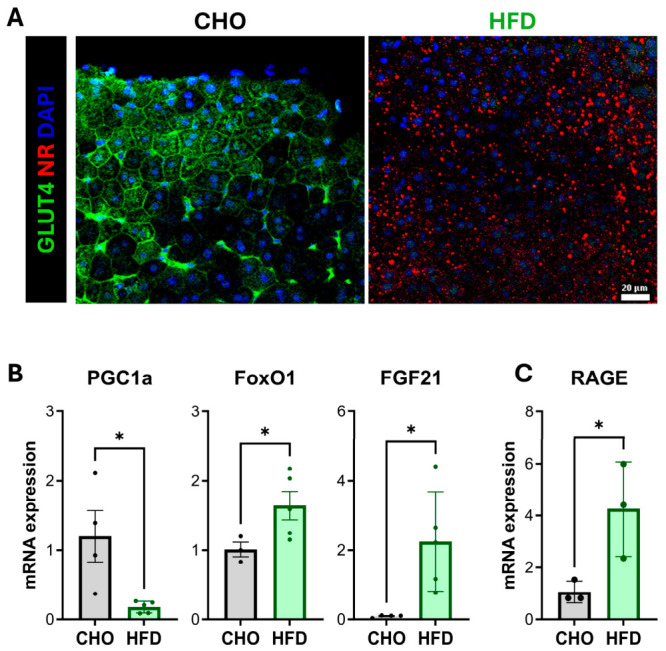
**Effects of HFD on liver metabolism.** (**A**) Immunofluorescent staining for GLUT4 and adipose content (NR—Nile Red) in control and HFD-treated livers (scale bar = 20 µm). (**B**) mRNA expression levels of genes related to liver metabolism. Mann–Whitney U test, * *p* < 0.05. (**C**) mRNA expression levels of AGE receptor (RAGE) in the liver. Student’s *t*-test, * *p* < 0.05.

The impaired glucose regulation and the development of insulin resistance (Figure 1) were supported by altered signaling. Immunofluorescence staining of whole-mount liver tissue revealed notable GLUT4 expression in hepatocytes from CHO-fed mice, while HFD-fed mice showed reduced GLUT4 expression (Figure 3A). Nile Red staining presents more lipid droplets in the livers of HFD-fed mice compared to CHO-fed mice (Figure 3A), consistent with the macro-view of adiposity (Figure 1C) and the histological results (Figure 2). To further assess the metabolic impact of a HFD, we analyzed the expression levels of key metabolic regulators in the liver using qPCR. PGC1α, a key transcriptional coactivator involved in mitochondrial biogenesis and energy metabolism, exhibited an 80% reduction in expression in the HFD group compared to the CHO-fed mice, showing impaired hepatic energy homeostasis. FoxO1, a key mediator of insulin signaling and hepatic control and a master regulator of metabolic homeostasis, along with FGF21, a hepatokine that induces increased fibrosis under metabolic stress, were significantly upregulated in HFD-fed mice. These molecular transcripts, suggesting enhanced stress adaptation in response to chronic metabolic overload, are consistent with them having a role in obesity and the development of insulin resistance (Figure 3B). RAGE, a receptor for advanced glycation end-products (AGEs) in HFD mice, was upregulated in HFD mice, reflecting increased AGE accumulation in the liver, induced by the diet (Figure 3C). Altogether, the reduced GLUT4 levels, elevated RAGE expression, and increased glucose levels are consistent with the development of insulin resistance and the appearance of AGEs.

Notably, HFD-fed mice exhibited impaired glucose tolerance, increased body weight, and elevated hepatic lipid accumulation and fibrosis, prompting further investigation into liver ECM remodeling. Given these metabolic alterations, we investigated the structural impact of HFDs on the hepatic ECM proteins. The alterations in the ECM under HFD nutrition suggest significant remodeling and a potential role for AGEs, which are closely associated with glycation-driven modifications of ECM proteins, particularly collagen, the major protein composing the organ scaffold. We aimed to explore how HFD-induced metabolic stress alters the liver ECM composition and structure. To further characterize these changes, we performed decellularization to isolate and analyze the liver ECM scaffold, providing deeper insight into ECM modifications under metabolic dysfunction.

**Figure 4:**
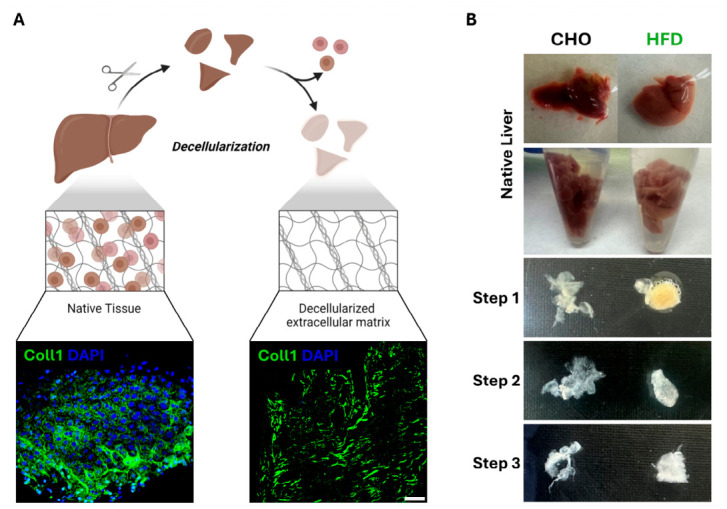
**Liver decellularization process in CHO- and HFD-treated mice.** (**A**) Schematic illustration of liver decellularization and immunofluorescence staining for collagen 1 (Coll1) and DAPI before and after decellularization (lower panel, scale bar = 20 µm). (**B**) Representative images of the native liver tissue through the distinct stages of decellularization in CHO- and HFD-treated groups.

The schematic illustration in Figure 4 depicts the decellularization (dECM) procedure, allowing further investigation of both physical and protein-level alterations induced by diet. Immunostaining with anti-collagen I highlighted the collagen fiber organization, and DAPI was used to stain the cell nuclei (Figure 4A). Following decellularization, cellular components were depleted, leaving a collagen-rich scaffold. The dECM was isolated (as described in the Materials and Methods Section and Figure 4B) to evaluate the changes in ECM induced by a HFD. The dECM structure and composition were assessed for protein modifications and their spatial distribution. Macroscopically, the livers from HFD mice appeared yellowish due to the accumulation of lipids (Figure 1C). Following decellularization, the resulting dECM from HFD livers appeared more solid, dense, and uniformly structured compared to that from CHO-fed mice (Figure 4). Throughout the stages of decellularization, the ECMs from HFD mice were consistently denser and more abundant compared to those from CHO-fed mice, and the isolated dECMs from CHO-fed mice were white, loosely organized, and fragile (Figure 4B).

**Figure 5:**
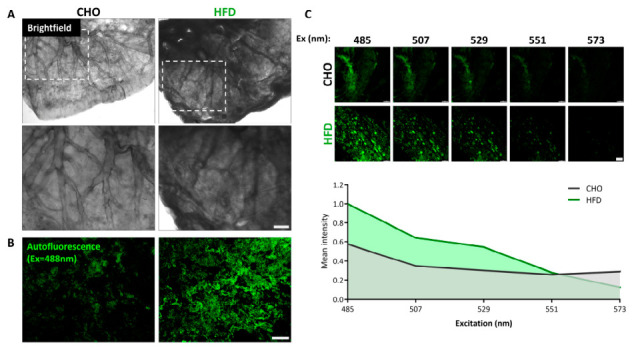
**Liver dECM architecture and autofluorescence properties.** (**A**) Liver dECM derived from CHO- and HFD-treated mice visualized by bright-field microscopy at low magnification (upper panel) and higher magnification (middle panel; ×200, scale bar = 300 µm). (**B**) Visualization by STELLARIS confocal of dECM autofluorescence of control and HFD groups after excitation with a 488 nm laser (scale bar = 50 µm). (**C**) Continuous spectrum scanning from 485 to 575 nm, with representative images of the emission pattern at different excitation wavelengths (scale bar = 20 µm).

Bright-field views of dECMs showed marked differences in morphology and organization between the diet groups (Figure 5A). dECMs from HFD-fed mice appeared denser and less transparent than those from CHO-fed controls. In contrast, the dECMs from CHO-fed mice were more translucent and displayed a distinct vascular network. These findings suggest that a HFD induces structural alterations in the liver ECM. Protein glycation affects intrinsic fluorescence, indicating AGE accumulation leading to cross-linking within proteins and collagen fibers, resulting in an increase in stiffness. We assessed intrinsic dECM fluorescence using the STELLARIS confocal platform, capturing both fluorescence imaging and emission spectra through comprehensive measurements. The microscope uses a white light laser (WLL) with continuous spectral output across the full spectrum, enabling the visualization of unstained samples based on glycation-induced changes in their intrinsic fluorescence. In Figure 5B, dECMs from HFD mice emitted a higher auto-fluorescence signal (excitation = 488 nm) compared to CHO-fed mice, reflecting glycation-associated spectral alterations. In Figure 5C, we visualize the dECM autofluorescence; this spectroscopy measurement is indicative of AGE accumulation, which was observed across a continuous excitation range from 485 to 573 nm, resulting in a two-fold increase in autofluorescence levels in dECM retrieved from HFD-fed mice compared to CHO-fed controls.

**Figure 6:**
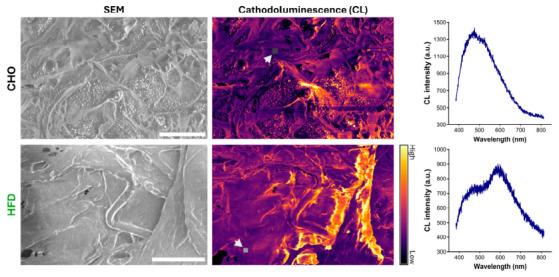
**dECM architecture view by SEM–cathodoluminescence spectroscopy.** dECM derived from CHO and HFD visualized by SEM (scale bar: CHO = 10 µm; HFD = 50 µm) and auto-cathodoluminescence (CL) signal mapping. Point CL spectral properties from CHO (upper panel)- and HFD (lower panel)-fed mice dECM (measured region marked by an arrow).

As shown in Figure 6, the SEM-CL analysis provides high-resolution imaging combined with simultaneous spectral data acquisition at nanometer-scale resolution. This integrated approach allowed for the detailed identification of protein molecular alterations, correlating structural and optical changes that point to glycation-induced modifications in the ECM. The dual imaging of SEM-CL enabled the localization and characterization of chemically modified regions, indicative of altered ECM remodeling under HFD nutrition. From SEM images, we observe that collagen fibers in the dECM from CHO mice possess a fine structure, while those from HFD-fed mice appeared thicker, due to glycation-induced crosslinking and intertwining, resulting in the formation of dense, mesh-like bundles (Figure 6, left panel). The hyperspectral auto-cathodoluminescence (auto-CL) with spatial imaging further supported these changes (Figure 6, middle panel). The CL properties, combined with SEM capabilities, enabled the correlation of optical properties with the structures and compositional features at the atomic scale. These nano-resolution images and spectral analysis of dECM samples compare structural and chemical features between CHO- and HFD-fed mice. The CHO-fed mice present a luminescence peak at around 400 nm, while samples from HFD mice had a broader emission spectrum, with notable luminescence peaks ranging from 400 nm to 600 nm (Figure 6, right panel). The spectral shift occurred due to significant chemical and compositional alterations within the ECM of HFD mice, consistent with the presence of AGEs. The novel information presented on the ECM arrangement enabled us to evaluate the structural and chemical changes.

**Figure 7:**
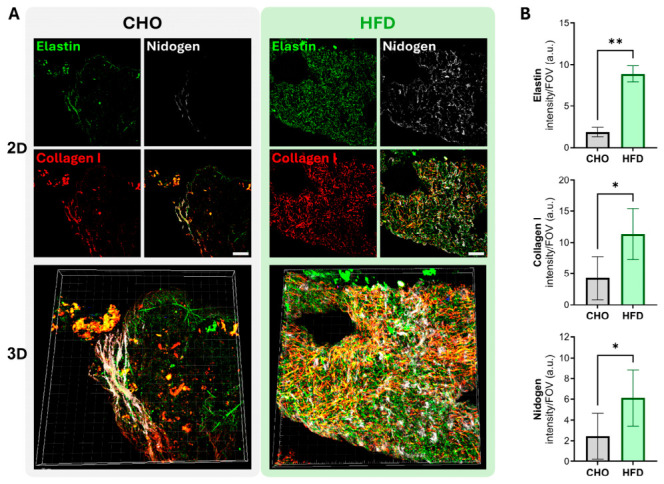
**ECM protein expression and 3D architecture.** (**A**) Immunofluorescence staining of elastin, collagen I, and nidogen in dECM from control and HFD mice, presented in 2D (upper panel; scale bar = 30 µm) and 3D reconstruction (lower panel), visualized using a spinning-disk confocal microscope. (**B**) Quantification of elastin, collagen I, and nidogen staining intensity. Data presented as mean ± SD, FOV—field of view. Student’s *t*-test * *p* < 0.05; ** *p* < 0.01.

Furthermore, immunostaining of dECM for collagen I, elastin, and nidogen proteins visualized their organization using spinning-disk confocal fluorescence microscopy. Fibers in dECM from HFD-fed mice appeared thicker and showed higher fluorescence intensity in both 2D and 3D imaging compared to those from CHO-fed mice, as shown in Figure 7A, and quantified intensity, as summarized in Figure 7B. The altered appearance of the ECM in HFD mice complements the morphological changes observed across multiple imaging modalities from histology (Figure 2), bright-field and confocal microscopy (Figure 5), and immunostaining (Figure 7) to nanoscale imaging by SEM-CL (Figure 6), collectively supporting structural remodeling of the liver ECM.

**Figure 8:**
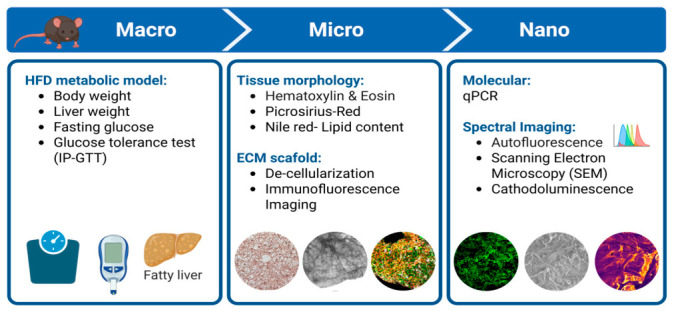
Schematic overview of the methods used in the analysis, along with scale.

**In summary**, nutritional factors such as a high-fat diet promote hyperglycation, resulting in chemical, physical, and structural alterations of ECM proteins from macro- to nano-resolution (Figure 8). The notable changes in ECM organization resulting from PTMs affect the scaffold of the liver, influence the cellular niche, and disrupt signaling pathways. The novel view of this study is the use of multiple spectroscopy imaging approaches to assess ECM protein glycation, i.e., AGEs formed under HFD nutrition. Autofluorescence imaging, confocal microscopy, and CL spectroscopy confirmed the accumulation of AGEs and the increase in protein cross-linking. Collectively, these modifications link ECM remodeling to metabolic dysfunction and liver pathology, affecting its remodeling and regeneration.

## 4. Discussion

Diabetes is a metabolic disease associated with hyperglycemia that promotes AGE formation, leading to modifications in protein structure. Here, we investigated the effects of nutrition-induced hyperglycation on liver tissue. Isolated livers from mice were used to assess the changes in protein structure and organization that are associated with increased cellular adiposity in the liver. The changes in tissue structure affect the organ scaffolding and its potential for regeneration.

Our earlier study demonstrated that HFD nutrition induces AGE formation and ECM remodeling in adipose tissue, in addition to altering body and tissue weight and ECM stiffness [16]. Similarly, elevated levels of AGEs have been observed in the liver under a HFD [22]. We prove that an accumulation of AGEs is associated with the upregulation of RAGE. The interaction between AGEs and RAGE in the liver ECM contributes to tissue fibrosis, potentially explaining the observations presented in this study and also reported elsewhere [23]. The AGEs formed under diet-induced obesity result from hyperglycemia and contribute to alterations in organ scaffolding, also leading to a change in cell signaling. Consequently, hyperglycemia is associated with increased protein crosslinking, leading to tissue stiffness in vivo [16,24,25,26] and in cell cultures in vitro [27,28]. This is consistent with impaired mitochondrial biogenesis under chronic hyperglycemia, which drives increased mitochondrial reactive oxygen species production and subsequent oxidative modification of ECM proteins [3,15]. This connects the hepatic molecular phenotype identified in this study to the glycoxidative ECM structural changes documented by multi-scale imaging.

AGEs drive dynamic liver fibrosis under chronic metabolic stress; the RAGE-dependent pathway promoting fibrotic gene expression and collagen production [29,30]. Elevated serum AGE levels [31] have been linked to a greater severity of hepatic steatosis and fibrosis in NAFLD patients [32], and correlate with metabolic risk factors such as hyperglycemia and insulin resistance. Consistently, our findings of ECM crosslinking and stiffness in HFD-fed mice coincide and result in glycoxidation and matrix disorganization, reinforcing the role of AGEs in fibrotic remodeling in metabolic liver disease.

In this study, we introduced novel approaches to reveal glycation-related ECM spectral signatures by employing label-free imaging such as SEM-CL and confocal STELLARIS to identify alterations in protein modification. The SEM-CL enables nanometer-scale characterization of both topographical features and optical properties. The altered structure revealed increased fiber density and disorganized mesh-like bundles in the dECM of HFD fed mice, along with stronger CL signals, reflecting glycoxidation-induced protein crosslinking and alterations (Figure 6). The findings align with the reported capability of auto-cathodoluminescence to resolve spectral shifts in collagen fibrils due to changes in molecular environment and glycation status [33]. The spectral heterogeneity we saw supports the interpretation that glycation introduces structural and compositional diversity across ECM regions. Notably, the autofluorescence profiles obtained using STELLARIS confocal imaging showed a related pattern of enhanced signal intensity and fiber reorganization in HFD samples (Figure 5), corroborating the chemical modifications detected by SEM-CL. Unlike conventional imaging, the benefit of using SEM-CL is that it does not require staining, making it particularly suited for detecting subtle chemical alterations in native ECM architecture [34]. These techniques complement histological and immunofluorescence staining methods for tissue adiposity and fibrosis. The combined methods provided converging evidence for AGE-driven ECM modifications, underlining altered mechanical properties in metabolic liver disease.

It is well recognized that ECM crosslinking increases matrix stiffness and alters viscoelastic properties, creating aberrant mechanical signals that disrupt normal cell regulation and contribute to fibrosis accumulation [5,7,16,27]. Alterations in the physical properties of ECM fibers critically affect cell–matrix interactions, influencing cellular behavior, signaling pathways, and tissue homeostasis [8,9,35]. ECM crosslinking impacts structural integrity and viscoelasticity, providing deeper insights into the pathological roles of altered biomechanical cues associated with metabolic diseases and fibrosis development and their impact on potential organ regeneration [10,36]. Recent findings have identified AGE-mediated ECM crosslinking as a hallmark of liver cirrhosis, contributing to increased stiffness and impaired remodeling [37]. Our results demonstrate that alterations in ECM chemical composition and changes in fiber organization in HFD-fed mice align with these concepts, highlighting the relevance of ECM modifications in diet-induced metabolic dysfunction.

The HFD mouse model robustly recapitulates key features of metabolic liver disease; the multiple induced metabolic perturbations are associated with hyperglycemia and therefore affect the ECM remodeling. AGE accumulation was monitored through intrinsic autofluorescence spectral shifts, RAGE upregulation, and established glycation chemistry. The use of the methods described here provides data consistent with glycation-associated molecular changes, which can be used in the pharmacological targeting of the AGE–ECM axis and may be of relevance in the context of metabolic liver disease. The present study can serve as a preclinical stage to better resolve the structural consequences of AGE modulation at the ECM from the nanoscale level. The multi-scale imaging framework established here, combining autofluorescence spectroscopy, confocal microscopy, and SEM-CL, directly addresses this gap, enabling label-free spatial mapping of potential glycation-induced crosslinking and spectral shifts in native ECM architecture. This provides a platform for evaluating whether therapeutic interventions restore ECM fiber organization, reduce crosslink density, and normalize the optical signatures associated with AGE accumulation, outcomes that biochemical assays alone cannot capture as they are focused on structural ECM characterization, improving remodeling processes to evaluate the changes that occurred under NAFLD.

## 5. Conclusions

HFD nutrition is associated with dysregulated energy homeostasis and is a major cause of insulin resistance, obesity, and type-2 diabetes. AGE formation alters ECM proteins, triggering an abnormal cell niche. Within this context, the formation of AGEs in ECM proteins disrupts the scaffolding of an organ and therefore the cellular niche. Elevated AGEs levels alter cell–matrix and cell–cell interactions, thereby amplifying metabolic dysregulation and organ remodeling and regeneration. Understanding ECM protein modifications may provide an insight into the mechanisms that govern altered cell signaling and tissue-specific expression patterns. These ECM-driven changes compromise the structural and functional environment of cells, contributing to metabolic impairment, pathogenesis, and hampered remodeling and regeneration of tissues.

## Figures and Tables

**Table 1:** PCR primers.

Primer	Sequence (5′–3′)
Rplp0_F	AGATTCGGGATATGCTGTTGGC
Rplp0_R	TCGGGTCCTAGACCAGTGTTC
FOXO1 F	TCAAGGATAAGGGCGACAGC
FOXO1 R	TGTCCATGGACGCAGCTCTT
FGF21 F	GAGCTCTCTATGGATCGCCTCA
FGF21 R	TGGTTTGGGGAGTCCTTCTG
PGC1A F	GTGTGCTGTGTGTCAGAGTG
PGC1A R	CCAGAGCAGCACACTCTATGT
RAGE F	AACACAGCCCCCATCCAA
RAGE R	GCTCACCAACAGCTGAATG

## Data Availability

The original contributions presented in this study are included in the article. Further inquiries can be directed to the corresponding author(s).

## Abbreviations

AGEs	Advanced glycation end-products
CHO	Carbohydrate diet
CL	Cathodoluminescence
dECM	Decellularized extracellular matrix
ECM	Extracellular matrix
HFD	High-fat diet
NAFLD	Nonalcoholic fatty liver disease
PTM	Post-translational modification
RAGE	Advanced glycation end-products receptor

## References

[1] Guo L., Xiang W., Pan Z., Gu H., Jiang X. (2025). Post-translational modifications of collagen and its related diseases in metabolic pathways. Acta Pharm. Sin. B.

[2] Smuda M., Glomb M.A. (2013). Fragmentation pathways during maillard-induced carbohydrate degradation. J. Agric. Food Chem..

[3] Brownlee M. (2005). The pathobiology of diabetic complications: A unifying mechanism. Diabetes.

[4] Shinohara M., Thornalley P.J., Giardino I., Beisswenger P., Thorpe S.R., Onorato J., Brownlee M. (1998). Overexpression of glyoxalase-I in bovine endothelial cells inhibits intracellular advanced glycation endproduct formation and prevents hyperglycemia-induced increases in macromolecular endocytosis. J. Clin. Investig..

[5] Chaudhuri J., Bains Y., Guha S., Kahn A., Hall D., Bose N., Gugliucci A., Kapahi P. (2018). The Role of Advanced Glycation End Products in Aging and Metabolic Diseases: Bridging Association and Causality. Cell Metab..

[6] Rondeau P., Bourdon E. (2011). The glycation of albumin: Structural and functional impacts. Biochimie.

[7] Singh V.P., Bali A., Singh N., Jaggi A.S. (2014). Advanced glycation end products and diabetic complications. Korean J. Physiol. Pharmacol..

[8] Mariman E.C.M., Wang P. (2010). Adipocyte extracellular matrix composition, dynamics and role in obesity. Cell. Mol. Life Sci..

[9] Ahmed B., Sultana R., Greene M.W. (2021). Adipose tissue and insulin resistance in obese. Biomed. Pharmacother..

[10] Lin D., Chun T.H., Kang L. (2016). Adipose extracellular matrix remodelling in obesity and insulin resistance. Biochem. Pharmacol..

[11] Klaas M., Kangur T., Viil J., Mäemets-Allas K., Minajeva A., Vadi K., Antsov M., Lapidus N., Järvekülg M., Jaks V. (2016). The alterations in the extracellular matrix composition guide the repair of damaged liver tissue. Sci. Rep..

[12] Feng J., Gong Z., Yang J., Mo Y., Song F. (2025). Machine learning-based integration reveals reliable biomarkers and potential mechanisms of NASH progression to fibrosis. Sci. Rep..

[13] Lian C.Y., Zhai Z.Z., Li Z.F., Wang L. (2020). High fat diet-triggered non-alcoholic fatty liver disease: A review of proposed mechanisms. Chem. Biol. Interact..

[14] Twarda-clapa A., Olczak A., Białkowska A.M., Koziołkiewicz M. (2022). Advanced Glycation End-Products (AGEs): Formation, Chemistry, Classification, Receptors, and Diseases Related to AGEs. Cells.

[15] Santos J.C.d.F., Valentim I.B., De Araújo O.R.P., Ataide T.D.R., Goulart M.O.F. (2013). Development of nonalcoholic hepatopathy: Contributions of oxidative stress and advanced glycation end products. Int. J. Mol. Sci..

[16] Naftaly A., Kislev N., Izgilov R., Adler R., Silber M., Shalgi R., Benayahu D. (2022). Nutrition Alters the Stiffness of Adipose Tissue and Cell Signaling. Int. J. Mol. Sci..

[17] Zeng X.-F., Varady K.A., Wang X.-D., Targher G., Byrne C.D., Tayyem R., Latella G., Bergheim I., Valenzuela R., George J. (2024). The role of dietary modification in the prevention and management of metabolic dysfunction-associated fatty liver disease: An international multidisciplinary expert consensus. Metabolism.

[18] Livak K.J., Schmittgen T.D. (2001). Analysis of relative gene expression data using real-time quantitative PCR and the 2^−ΔΔCT^ Method. Methods.

[19] Kleiner D.E., Brunt E.M., Van Natta M., Behling C., Contos M.J., Cummings O.W., Ferrell L.D., Liu Y.-C., Torbenson M.S., Unalp-Arida A. (2005). Design and validation of a histological scoring system for nonalcoholic fatty liver disease. Hepatology.

[20] Baptista P.M., Siddiqui M.M., Lozier G., Rodriguez S.R., Atala A., Soker S. (2011). The use of whole organ decellularization for the generation of a vascularized liver organoid. Hepatology.

[21] Kislev N., Izgilov R., Adler R., Benayahu D. (2021). Exploring the cell stemness and the complexity of the adipose tissue niche. Biomolecules.

[22] Zhao Y., Wang P., Sang S. (2019). Dietary genistein inhibits methylglyoxal-induced advanced glycation end product formation in mice fed a high-fat diet. J. Nutr..

[23] Goodwin M., Herath C., Jia Z., Leung C., Coughlan M.T., Forbes J., Angus P. (2013). Advanced glycation end products augment experimental hepatic fibrosis. J. Gastroenterol. Hepatol..

[24] Naemi R., Romero Gutierrez S.E., Allan D., Flores G., Ormaechea J., Gutierrez E., Casado-Pena J., Anyosa-Zavaleta S., Juarez M., Casado F. (2022). Diabetes Status is Associated With Plantar Soft Tissue Stiffness Measured Using Ultrasound Reverberant Shear Wave Elastography Approach. J. Diabetes Sci. Technol..

[25] Wang W., Hapach L.A., Griggs L., Smart K., Wu Y., Taufalele P.V., Rowe M.M., Young K.M., Bates M.E., Johnson A.C. (2022). Diabetic hyperglycemia promotes primary tumor progression through glycation-induced tumor extracellular matrix stiffening. Sci. Adv..

[26] Fan W., Adebowale K., Váncza L., Li Y., Rabbi M.F., Kunimoto K., Chen D., Mozes G., Chiu D.K.C., Li Y. (2024). Matrix viscoelasticity promotes liver cancer progression in the pre-cirrhotic liver. Nature.

[27] Izgilov R., Naftaly A., Benayahu D. (2023). Advanced Glycation End Products Effects on Adipocyte Niche Stiffness and Cell Signaling. Int. J. Mol. Sci..

[28] Ramasamy R., Yan S.F., Schmidt A.M. (2011). Receptor for AGE (RAGE): Signaling mechanisms in the pathogenesis of diabetes and its complications. Ann. N. Y. Acad. Sci..

[29] He Y.L., Zhu J.Q., Huang Y.Q., Gao H., Zhao Y.R. (2015). Advanced glycation end product (AGE)-induced hepatic stellate cell activation via autophagy contributes to hepatitis C-related fibrosis. Acta Diabetol..

[30] Tabar M.S., Nilghaz M., Hekmatdoost A., Pashayee-Khamene F., Mokhtari Z., Karimi S., Ahmadzadeh S., Saberifiroozi M., Hatami B., Yari Z. (2025). Advanced glycation end products and risk of mortality in patients with cirrhosis: A prospective cohort study. Sci. Rep..

[31] Naftaly A., Izgilov R., Omari E., Benayahu D. (2021). Revealing Advanced Glycation End Products Associated Structural Changes in Serum Albumin. ACS Biomater. Sci. Eng..

[32] Pereira E.N.G.D.S., Paula D.P., Araujo B.P., Fonseca M.J.M.D., Diniz M.F.H.S., Daliry A., Griep R.H. (2021). Advanced glycation end product: A potential biomarker for risk stratification of non-alcoholic fatty liver disease in ELSA-Brasil study. World J. Gastroenterol..

[33] Zielinski M.S., Vardar E., Vythilingam G., Engelhardt E.M., Hubbell J.A., Frey P., Larsson H.M. (2019). Quantitative intrinsic auto-cathodoluminescence can resolve spectral signatures of tissue-isolated collagen extracellular matrix. Commun. Biol..

[34] Thiberge S., Nechushtan A., Sprinzak D., Gileadi O., Behar V., Zik O., Chowers Y., Michaeli S., Schlessinger J., Moses E. (2004). Scanning electron microscopy of cells and tissues under fully hydrated conditions. Proc. Natl. Acad. Sci. USA.

[35] Conway J.R.W., Isomursu A., Follain G., Härmä V., Jou-Ollé E., Pasquier N., Välimäki E.P.O., Rantala J.K., Ivaska J. (2023). Defined extracellular matrix compositions support stiffness- insensitive cell spreading and adhesion signaling. Proc. Natl. Acad. Sci. USA.

[36] Guo T., Wantono C., Tan Y., Deng F., Duan T., Liu D. (2023). Regulators, functions, and mechanotransduction pathways of matrix stiffness in hepatic disease. Front. Physiol..

[37] Lyu C., Kong W., Liu Z., Wang S., Zhao P., Liang K., Niu Y., Yang W., Xiang C., Hu X. (2023). Advanced glycation end-products as mediators of the aberrant crosslinking of extracellular matrix in scarred liver tissue. Nat. Biomed. Eng..

